# The E3 Ligase TRIM25 Impairs Apoptotic Cell Death in Colon Carcinoma Cells via Destabilization of Caspase-7 mRNA: A Possible Role of hnRNPH1

**DOI:** 10.3390/cells12010201

**Published:** 2023-01-03

**Authors:** Usman Nasrullah, Kristina Stanke, Victoria Recknagel, Süleyman Bozkurt, Patrick Wurzel, Stefan Gauer, Gergely Imre, Christian Münch, Josef Pfeilschifter, Wolfgang Eberhardt

**Affiliations:** 1 Institute of General Pharmacology and Toxicology, Goethe University Frankfurt, D-60590 Frankfurt, Germany; 2 Institute of Biochemistry II, Goethe University Frankfurt, D-60590 Frankfurt, Germany; 3 Frankfurt Institute for Advanced Studies, D-60590 Frankfurt, Germany; 4 Faculty of Medicine, Goethe University Frankfurt, D-60590 Frankfurt, Germany

**Keywords:** apoptosis, caspase-7, colon carcinoma cells, hnRNPH1, RNA-binding proteins, TRIM25

## Abstract

Therapy resistance is still a major reason for treatment failure in colorectal cancer (CRC). Previously, we identified the E3 ubiquitin ligase TRIM25 as a novel suppressor of caspase-2 translation which contributes to the apoptosis resistance of CRC cells towards chemotherapeutic drugs. Here, we report the executioner caspase-7 as being a further target of TRIM25. The results from the gain- and loss-of-function approaches and the actinomycin D experiments indicate that TRIM25 attenuates caspase-7 expression mainly through a decrease in mRNA stability. The data from the RNA pulldown assays with immunoprecipitated TRIM25 truncations indicate a direct TRIM25 binding to caspase-7 mRNA, which is mediated by the PRY/SPRY domain, which is also known to be highly relevant for protein–protein interactions. By employing TRIM25 immunoprecipitation, we identified the heterogeneous nuclear ribonucleoprotein H1 (hnRNPH1) as a novel TRIM25 binding protein with a functional impact on caspase-7 mRNA stability. Notably, the interaction of both proteins was highly sensitive to RNase A treatment and again depended on the PRY/SPRY domain, thus indicating an indirect interaction of both proteins which is achieved through a common RNA binding. Ubiquitin affinity chromatography showed that both proteins are targets of ubiquitin modification. Functionally, the ectopic expression of caspase-7 in CRC cells caused an increase in poly ADP-ribose polymerase (PARP) cleavage concomitant with a significant increase in apoptosis. Collectively, the negative regulation of caspase-7 by TRIM25, which is possibly executed by hnRNPH1, implies a novel survival mechanism underlying the chemotherapeutic drug resistance of CRC cells. The targeting of TRIM25 could therefore offer a promising strategy for the reduction in therapy resistance in CRC patients.

## 1. Introduction

Colorectal cancer (CRC) is one of the most common tumors in humans and among the most prevalent causes of cancer-related death worldwide [1,2]. Despite advances in surgery and implementation of novel pharmacological therapies, the frequent occurrence of therapy resistance strongly limits the benefit of current cancer therapeutics [3]. Consequently, novel therapeutic options which help to overcome the therapy resistance of tumor cells are warranted. Despite mutations in tumor suppressor genes, the impaired activation of apoptotic caspases critically contributes to cancerous progression and impaired sensitivity to chemotherapeutic drugs [4,5]. Although most caspases are constitutively expressed and predominantly regulated through activation of their inactive zymogens, we previously reported on the inhibition of the pro-apoptotic caspase-2 at the level of translation by the ubiquitin E3 ligase tri-partite motif-containing protein 25 (TRIM25) as a novel cell survival mechanism in human colon carcinoma cells [6].

Importantly, the previous reports unraveled the way that the functional effects of TRIM25, in addition to its ubiquitin-modifying activity, can result from direct regulation of nucleic acids [7,8]. Among the newly emerging class of RNA-binding E3 ligases confirmed by different human RNA interactome capture studies [9], TRIM25 is one with a broad pathophysiological relevance. Although lacking typical RNA-binding domains (RBDs), TRIM25 exerts a strong RNA-binding affinity which has mainly been assigned to the PRY/SPRY domain at the C-terminus of the protein [8]. In addition to its originally described role in antiviral innate immunity, TRIM25 can interfere with diverse oncogenic as well as tumor suppressive pathways, including the phosphoinositide-3 kinase (PI3), the transforming growth factor β (TGFβ), or p53, as reviewed in [10]. In breast cancer, TRIM25 represents a key tumorigenic factor, which, independently of estrogen signaling, is pivotally involved in the transcriptional and posttranscriptional control of metastatic gene signatures [11].

We searched for novel apoptosis regulatory proteins controlled by TRIM25 by using high-resolution quantitative proteomics. Thereby, we identified caspase-7 as a protein which was most explicitly upregulated upon TRIM25 silencing. Caspase-7 belongs to the subgroup of executioner caspases, which, for their activation, require cleavage at the conserved aspartic acid residues by initiator caspases-8 or -9, which are relevant for the cleavage of specific substrates during the late (demolition) phase of apoptosis [12]. Among the most prominent targets of caspase-7 to be mentioned is the DNA damage sensor poly ADP-ribose polymerase 1 (PARP-1). Here, the caspase-7-mediated inactivation of PARP is a shared feature of apoptotic and pyroptotic cell death [13]. Although caspase-3 and -7 share common peptide recognition sequences in their substrates and the functional redundancy, a growing body of experimental evidence indicates non-redundant functions and distinct roles of both executioner caspases in apoptosis and in non-apoptotic processes [14,15,16]. In the context of cancer, a downregulation of caspase-7 could represent a potential marker of colonic carcinoma [17]. In addition, several genetic polymorphisms in the caspase-7 gene affecting caspase-7 mRNA expression have been described in cancer development [18]. However, the mechanisms underlying the pathologic deregulation of caspase-7 expression have not been characterized in more detail. In the present study, we unveil the destabilization of caspase-7 mRNA as a novel mRNA modulatory function by TRIM25, which may functionally contribute to the pathological downregulation of caspase-7 observed in colon carcinoma [17]. The constitutive suppression of caspase-7 expression through the attenuation of mRNA stability highlights a novel mechanism of drug resistance in human carcinoma cells executed by the multifunctional E3 ligase TRIM25.

## 2. Materials and Methods

### 2.1. Reagents and Antibodies

The doxorubicin was from Biotrend Chemicals (Cologne, Germany). The actinomycin D (from the Streptomyces species), staurosporine, etoposide, rapamycin, and biotin maleimide were from Merck (Darmstadt, Germany). A list of the antibodies used in this study is provided in the “Supplementary information” section. The ECL system and hyperfilms were from GE Healthcare (Freiburg, Germany). The Go-Taq polymerase was from Promega (Mannheim, Germany). The RevertAid Reverse Transcriptase and RiboLock RNase Inhibitor were from ThermoFisher Scientific (Dreieich, Germany). All the cell culture media, supplements, and modifying enzymes were purchased from Invitrogen (Karlsruhe, Germany), if not otherwise indicated.

### 2.2. Cell Culture

The human colorectal carcinoma cell lines DLD-1, CaCo-2, and HCT-15 were obtained from the German Collection of Microorganisms and Cell Cultures (Braunschweig, Germany). The RKO and HEK293 cells were obtained from the American Type Culture Collection (LGC-Promochem, Wiesbaden, Germany). The cells were grown in Dulbecco’s modified Eagle’s medium, supplemented with 10% heat-inactivated fetal calf serum, 100 U/mL penicillin, and 100 µg/mL streptomycin.

### 2.3. Small Hairpin (sh)-Mediated Knockdown of TRIM25

A generation of stable TRIM25 knockdown cells using lentiviral shRNA particles was made. A stable knockdown of TRIM25 in RKO cells was achieved by MISSION Lentiviral Transduction Particles (SHCLNV-NM_005082) from Merck. The following clones were employed independently:

TRIM25 #1 (TRCN0000272649);

TRIM25 #2 (TRCN0000272699);

TRIM25 #3 (TRCN0000003449);

TRIM25 #4 (TRCN0000272650);

TRIM25 #5 (TRCN0000272698);

Non-target shRNA control transduction particles (SHC002V).

The procedure for lentiviral knockdown was performed as described previously [19]. The efficiency of the knockdown was validated by Western blot analysis.

### 2.4. Small Interference (si)RNA

The epithelial cells were plated on 6 cm dishes to obtain 30–40% confluency at the day of transfection. The subconfluent cells were transfected with siRNAs by using the Oligofectamine reagent (Invitrogen) by following the manufacturer’s instructions. Gene silencing was performed by the transfecting of siRNA duplexes from 50 nM of validated siRNAs against human TRIM25 (ID#15204, “siTRIM25#1”) from ThermoFisher Scientific or, alternatively, a mixture of FlexiTube siRNAs for human TRIM25 (SI0000072170, SI0000072163, SI0000072156 and SI0000072149, “siTRIM25#2”) from Qiagen (Hilden, Germany). Alternatively, the cells were transfected with the same amounts of FlexiTube siRNA duplexes for hnRNPH (“sihnRNPH#1”) from Dharmacon (St. Leon-Rot, Germany) or from Santa Cruz (“sihnRNP#2”). A non-targeting control siRNA (siCtrl, #D001206-13) was from Dharmacon. The specific silencing of the targeted genes was validated by Western blot analysis.

### 2.5. Ectopic Expression of Plasmids in RKO Cells

The subconfluent RKO cells were grown on 60 mm dishes and transiently transfected with 6 µg of pFLAG-CMV-TRIM25 (Addgene plasmid #12449) [20] and pcDNA3-caspase-7-Flag (Addgene plasmid #11815) [21] or, alternatively, with the same amount of the corresponding empty vector by using the Lipofectamine 2000 reagent (ThermoFisher Scientific). Routinely, 48 h after transfection, the cells were used for further applications.

### 2.6. Western Blot Analysis

Whole cell homogenates were prepared as described previously [22] Briefly, the cells were lysed in ice-cold lysis buffer (137 mM NaCl, 20 mM Tris-HCl pH 8.0, 5 mM EDTA pH 8.0, 10% glycerol, and 1% Triton X-100) supplemented with a protease inhibitor mix (Roche, Mannheim, Germany) by several freeze–thaw cycles and cell debris separated by centrifugation for 30 min at 11.000× *g*. Subsequently, the supernatants were mixed with 4× Laemmli buffer (40% Glycerol, 10% SDS, 125 mM Tris-HCl pH 6.8, 50 mM DTT, 0.01% Bromphenol blue), heated for 5 min at 95 °C, and resolved by 8%-15% SDS polyacrylamide gel electrophoresis. The separated proteins were transferred for immunodetection onto nitrocellulose membranes (LC2003, ThermoFisher Scientific), and the membranes were blocked for 1 h in 5% milk in TBST (20 mM Tris-HCl pH 7.5, 150 mM NaCl, 0.1% Tween) at room temperature before the membranes were successively incubated with the specific primary and secondary antibodies listed in the “Supplementary information” section. The immunopositive signals were visualized with chemiluminescence using an ECL system from GE Healthcare. The quantification of the Western blot detection was performed by using the ImageJ software.

### 2.7. Isolation of Nuclear and Cytoplasmic Fractions

Nuclear “miniextracts” from colon carcinoma cells were prepared by following a protocol from Schreiber et al. [23]. Briefly, the cells were scraped in ice-cold PBS/5 mM EDTA and collected by centrifugation for 5 min at 5.000× *g* before the cell pellets were resuspended in hypotonic buffer A (10 mM Hepes, pH 7.9, 10 mM KCl, 0.1 mM EDTA, 0.1 mM EGTA, 1 mM Na_3_VO_4_, 1 mM NaF) supplemented with a protease inhibitor mix (Roche). Subsequently, the lysates were kept on ice for 10 min before 1/16th of buffer A’s volume of a 10% solution of Nonidet NP40 was added, and the cell lysates were vortexed for 10 s before centrifuged for 3 min at 7.000× *g*. The supernatants were collected as crude “cytoplasmic fractions” and stored at 20 °C. The pellets were resuspended in high-salt buffer C (20 mM Hepes, pH 7.9, 25% glycerol, 400 mM NaCl, 1 mM EDTA, 1 mM EGTA, 1 mM Na_3_VO_4_, 1 mM NaF) supplemented with a protease inhibitor mix (Roche) and vigorously rocked for 30 min on a shaker machine at 4 °C. After a final centrifugation at 11.000× *g* for 30 min, the supernatants were collected and stored as crude “nuclear extracts” at −20 °C.

### 2.8. qRT-PCR-Analysis

The total RNA was extracted from whole cells by using the Tri reagent (Merck) following the instructions of the manufacturer. First strand cDNA from equal amounts of RNA was synthesized by using random hexamer primer and the RevertAid RT Reverse Transcription Kit. The mRNA levels for the specific genes were determined by using a protocol according to the ‘hot start’ real-time PCR procedure with gene-specific TaqMan probes: TRIM25: Hs01116121_m1, Casp7: Hs00169152_m1, hnRNP H1: Hs01033848_m1, 18S: Hs99999901_s1, all from ThermoFisher Scientific. The assay mix 2× qPCRBIO Probe Mix Lo-ROX was purchased from Nippon Genetics (Düren, Germany). The C(T) values were normalized to the C(T) values of 18S mRNA within the same sample and quantified by using the 2^−ΔΔCt^ method.

### 2.9. Sub-G1 Analysis by Flow Cytometry

The analysis of the cell cycle distribution of the colorectal carcinoma cells was performed with a FACSCanto II flow cytometer (Becton Dickinson, Heidelberg, Germany), as described previously [24]. Briefly, the cells were trypsinized, washed in PBS, and fixed overnight in absolute ethanol at −20 °C. After centrifugation (300× *g* for 2 min), the cell pellets were resuspended in 0.3 mL hypotonic buffer containing 50 µg/mL propidium iodide (Merck); 0.1% sodium citrate; 0.1% Triton X-100; and 10 µg/mL RNase A and incubated for 30 min at 37 °C before measurement. The cells were gated to exclude cell debris and analyzed by flow cytometry in linear mode by using the FACSDiva Software (Becton Dickinson).

### 2.10. Immunoprecipitation of Endogenous Proteins

For immunoprecipitation (IP) of the endogenous proteins, the total cell lysates (300–500 µg) were precleared for 2 h at 4 °C with protein G-Sepharose 4 Fast Flow (GE Healthcare). The precleared lysates were incubated overnight at 4 °C, either with 2 µg of the IP antibody or, alternatively, with the same amount of species-specific IgG in a lysis buffer (137 mM NaCl, 20 mM Tris-HCl pH 8.0, 5 mM EDTA pH 8.0, 10% glycerol, 1% Triton X-100, 1 mM Na_3_VO_4_, 1 mM NaF, protease inhibitor mix) plus 5% fetal calf serum in a total volume of 1 mL. The samples were supplemented with Ribolock (final concentration: 100 U/mL) to minimize degradation of the bridging mRNA. The next day, 70 µL of fresh protein G-Sepharose beads were added and incubated at 4 °C for another 2 h with continuous rotation. After centrifugation for 2 min at 3000× *g*, the antigen–antibody-bound beads were washed three times with a low-salt buffer (50 mM Tris-HCl pH 7.5, 150 mM NaCl, 0.2% Triton X-100, 2 mM EDTA, 2 mM EGTA, 0.1% SDS, 100 U/mL Ribolock) and three times with a high-salt buffer (50 mM Tris-HCl pH 7.5, 500 mM NaCl, 0.2% Triton X-100, 2 mM EDTA, 2 mM EGTA, 0.1% SDS, 100 U/mL Ribolock), respectively. After the last washing step, the beads were dried and dissolved in 15–25 µL of Laemmli buffer and boiled for 10 min at 95 °C before the samples were loaded on SDS-PAGE gels.

### 2.11. Immunoprecipitation of Flag-Tagged Proteins

The IP of the Flag-tagged proteins and coprecipitating proteins or RNA was accomplished by using monoclonal anti-Flag-M2 antibodies coupled to magnetic beads (Merck). To reduce unspecific RNA binding, for the RNA-IP, the beads were preincubated with 0.5 mL of tRNA (10 µg tRNA/mL TBS) for 1 h at 4 °C under constant rotation. Thereafter, the beads were washed three times with 0.5 mL TBS (20 mM Tris-HCl pH 7.5, 150 mM NaCl). Forty microliters of preconditioned beads were added to 300–500 µg of cell lysates and the volume was adjusted to 1 mL with a lysis buffer (137 mM NaCl, 20 mM Tris-HCl pH 8.0, 5 mM EDTA pH 8.0, 10% glycerol, 1% Triton X-100, 1 mM Na_3_VO_4_,10 mM NaF, and a protease inhibitor mix) and incubated overnight at 4 °C under rotation. Subsequently, the beads were washed five times with 0.5 mL of TBS buffer. After the final washing step, the wash buffer was completely removed, the dried beads were dissolved in 15–25 µL of Laemmli buffer and boiled for 10 min at 95 °C, and the samples were resolved by SDS-PAGE.

### 2.12. Ribonucleoprotein (RNP) IP RT-PCR Assay

The Flag-tagged or endogenous RNA-binding proteins were immunoprecipitated as described before. The dried beads were dissolved in 1 mL of Tri reagent (Merck), and the precipitated RNA was purified by following the manufacturer´s instructions. Two hundred nanograms of RNA transcripts were reverse transcribed (RT) using random hexamer primer and the RevertAid RT Reverse Transcription Kit (ThermoFisher Scientific) before being analyzed by semiquantitative RT-PCR using Go-Taq hot start polymerase (Promega). The following primer sets were used: Caspase-7 forward: 5′- AGT GAC AGG TAT GGG CGT TC-3′; Caspase-7 reverse: 5′- CGG CAT TTG TAT GGT CCT CT-3′; GAPDH forward: 5′-CAC CAT CTT CCA GGA GCG AG; and GAPDH reverse: 5′-GCA GGA GGC ATT GCT GAT-3′. The PCR products were separated on a 1% agarose gel containing GelRed from Biotium (Biotrend, Cologne, Germany). The equality of the input RNA was confirmed by the RT reaction of the total cellular RNA isolated from 25 µg of cell extract used for IP and the subsequent assessment of the input mRNA level.

### 2.13. Construction of Flag-Tagged TRIM25 Truncations

The full-length vector pFlag-CMV2-EFP (pFlag-CMV2-TRIM25) for ectopic overexpression of TRIM25 was purchased from Addgene [20]. The TRIM25 truncations TRIM25 ΔRING (aa 57-630) and TRIM25 ΔPRY/SPRY (aa 1-457) were generated using full-length TRIM25 as a template. The TRIM25 deletions were amplified with the use of the KAPA HiFi HotStart ReadyMixPCR Kit (Kapa Biosystems, Merck) and the following primer bearing restriction sites for EcoRI (forward) and XbaI (reverse) were used: ΔRING forward: 5′-ATA TGA ATT CAT ACC AGG CGC GAC CGC AGC TG-3′; ΔRING reverse: 5′-ATA TTC TAG ACT ACT TGG GGG AGC AGA TGG A-3′; ΔPRY/SPRY forward: 5′-ATA TGA ATT CAG CAG AGC TGT GCC CCC TGG CC-3′; and ΔPRY/SPRY reverse: 5′-TAT TCT AGA CTA AAT GTA ATA CTC CAG GAG CTC-3′. The length of the digested amplicons was confirmed by agarose gel electrophoresis and subsequently, the gel-extracted bands were ligated with the EcoRI/XbaI-cut pFlag-CMV2 vector using T4 DNA Ligase from ThermoFisher Scientific. The correct sequences of all the constructs were confirmed by Sanger Sequencing (Microsynth Seqlab, Göttingen, Germany).

### 2.14. Generation of a Plasmid Bearing the 3′UTR of Caspase-7 for In Vitro Transcription

For the generation of pCR2.1-Topo-3′-UTR-caspase-7, the TOPO™ TA Cloning™ Kit (Invitrogen) was used by following the manufacturer´s instructions. The cDNA from the RKO cells was amplified by using HotStart Taq polymerase (ThermoFisher Scientific) and the following primers: 3′-UTR-caspase-7 forward: 5′-TAT TCT AGA GGG TAT TGA GTG TGA TTT GAA TGA TTT TTC ATT GGC-3′ and 3′-UTR-caspase-7 reverse: 5′-TAT GGC CGG CCT GGC TCC ATT TTC CAC AAT CCA TTG GT-3′.

### 2.15. RNA Affinity Chromatography

RNA affinity chromatography (Biotin pull down assay) was performed as described previously [6]. A biotinylated RNA sense probe (based on NM_033340.4) was generated by using 10 µg of EcoRI-linearized plasmid pCR2.1-Topo-3′-UTR-caspase-7 with the help of the “RiboMax-Large Scale RNA Production System-T7” (Promega, Mannheim, Germany). The eluted pulldown material and equal input levels were analyzed and confirmed by Western blotting.

### 2.16. Mass Spectrometry (LC-MS)

The sample preparation for mass spectrometry was performed as described previously [25]. Briefly, the cells were lysed and denatured as described for the Western blot analysis, but in contrast to mixing the samples with Laemmli buffer, the pure proteins were obtained by employing methanol/chloroform precipitation. The protein pellets were then resuspended in 8% urea/10 mM EPPS, pH 8.2. One hundred micrograms of protein per sample was digested overnight at 37 °C with LysC (FUJIFILM Wako Chemicals, Neuss, Germany) at a 1:50 (*w/w*) ratio and Trypsin (Promega, V5113) at 1:100 (*w/w*). The digested peptides were purified using Sep-Pak tC18 cartridges (Waters, Cooperation, Milford, MA USA). The peptide concentrations were determined with a μBCA assay (ThermoFisher Scientific, # 23235), and 10 μg of peptide per sample was labeled with Tandem Mass Tag (TMT) reagents (A34808, Thermo Scientific). The TMT-labeled samples were adjusted to equal amounts and pooled. The peptides were analyzed by Multi-Notch MS3-based TMT method [26] for total proteomics. The experimental details of the analysis of the raw data are provided in the “Supplementary information” section. The mass spectrometry proteomics data have been deposited to the ProteomeXchange Consortium with the use of the PRIDE database [27].

### 2.17. Indirect Immunofluorescence Microscopy

The analysis of the intracellular TRIM25 and hnRNPH1 distribution was performed by confocal microscopy, as described in [28]. Briefly, the cells which were plated on microscopic chamber slides (ibidiTreat) from Ibidi GmbH (Gräfelfing, Germany) were washed with ice-cold PBS and exposed to ice-cold 4% (*w/v*) paraformaldehyde/PBS pH 7.2 plus 0.25% (*w/v*) Triton X-100 in PBS for 15 min for fixation and permeabilization before being washed with ice-cold PBS. Subsequently, the slides were blocked for 30 min in Roti block (Carl Roth, Germany). Double staining was performed by incubating the cells with two antibodies simultaneously overnight at 4 °C. A polyclonal anti-hnRNPH1 antibody (ab10374, Abcam) and a monoclonal anti-TRIM25 antibody (sc-166926, Santa Cruz Biotechnology) were used. Both antibodies were used at a concentration of 1:100 in PBS. Subsequently, the cells were washed with PBS/Tween20 and incubated for 30 min with anti-mouse DyLight488-coupled and anti-rabbit DyLight550-coupled secondary antibodies (Thermo Fisher Scientific) and thereafter washed again with PBS. The cell nuclei were stained with 4,6-diamidino-2-phenylindole. The fluorescence was detected and documented using a Leica confocal microscope (SP8).

### 2.18. Statistical Analysis

The data are given as means ± SD. For statistical analysis, the unpaired two-tailed *t*-test was applied using GraphPad Prism version 8 (GraphPad Software, Inc., La Jolla, CA, USA). A *p* value ≤ 0.05 was considered significant. The sample size (n) is indicated in the figure legends.

## 3. Results

### 3.1. Identification of Caspase-7 as a Negative Target of TRIM25

**Figure 1:**
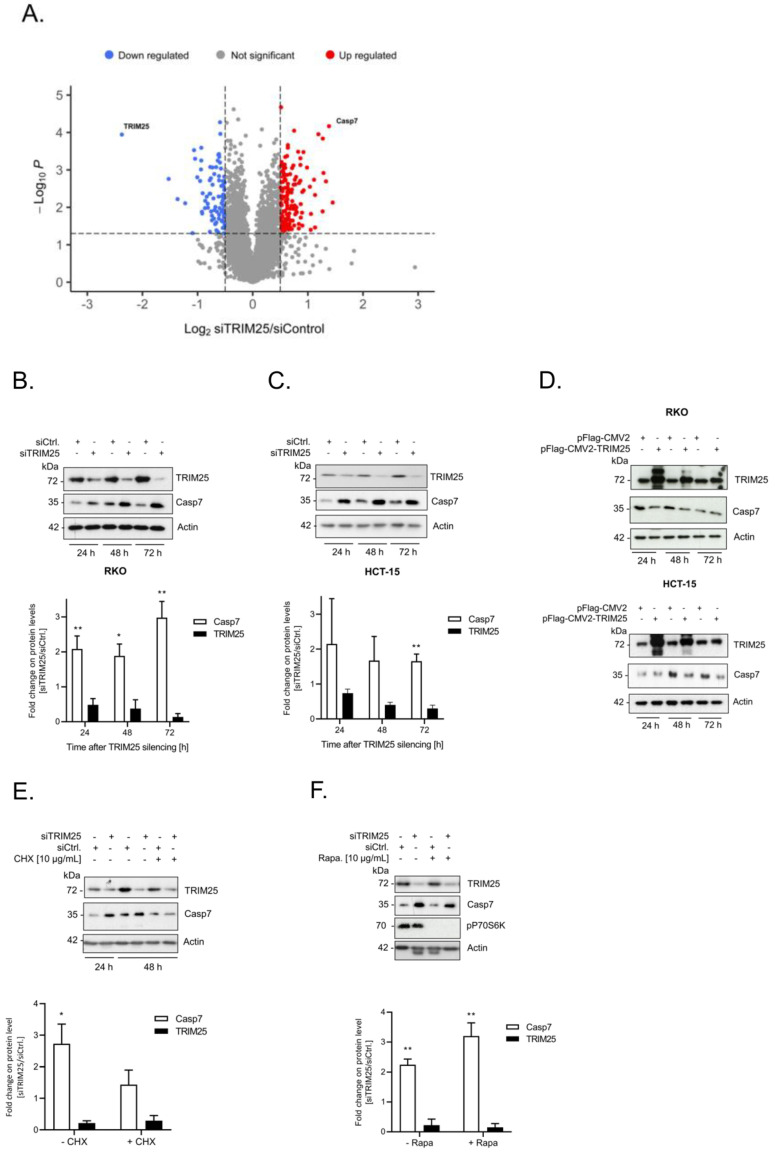
(**A**–**D**). Silencing of TRIM25 is concomitant with elevated caspase-7 protein level. (**A**) Volcano plot diagram showing LC/MS data from the analysis of RKO cells transfected for 48 h with control siRNA duplexes (siCtrl.) versus cells transfected with siRNA duplexes of TRIM25 (n = 4). Colored dots depict significantly (*p* ≤ 0.01) upregulated (red) and downregulated (blue) proteins (fold changes ≥ or ≤ 0.5-fold). For clarity, only the positions of TRIM25 and caspase-7 are shown. (**B**,**C**). Time-dependent changes in caspase-7 protein level after TRIM25 knockdown. Subconfluent RKO (**B**) or HCT-15 (**C**) cells were transfected with control siRNA duplexes (siCtrl.) or with siRNA duplexes of TRIM25 (siTRIM25) for the indicated time periods before the content of caspase-7 (Casp7) was monitored by Western blot analysis with β-actin (Actin) as a loading control. Data in the lower panels represent means ± SD (n = 3) * *p* ≤ 0.05, ** *p* ≤ 0.01 caspase-7 contents in siTRIM25 vs. control siRNA transfectants, which were set as one-fold. (**D**) Time-dependent changes in caspase-7 protein levels after ectopic TRIM25 expression. Subconfluent RKO (upper panel) or HCT-15 (lower panel) cells were transfected either with 6 µg of empty pCMV-Flag vector (pFlag-CMV2) or, alternatively, with the same amount of Flag-tagged human TRIM25 (pFlag-CMV2-TRIM25) for the indicated time periods before the content of caspase-7 was monitored by Western blot analysis. (**E**,**F**). The increase in caspase-7 upon TRIM25 knockdown does not result from increased caspase-7 protein stability. Subconfluent RKO cells were transfected with siRNA duplexes of TRIM25 (siTRIM25) or control siRNA duplexes (siCtrl.) for 24 h before translation was blocked by the addition of (**E**) 10 µg/mL cycloheximide (+CHX) or, alternatively, with (**F**) 100 ng/mL of rapamycin (Rapa.) for a further 24 h. Thereafter, the cells were harvested and extracted for total protein lysates for analysis of TRIM25, caspase-7, or, additionally, for phospho-p70S6 kinase (**F**). Graphs show means ± SD (n = 3) * *p* ≤ 0.05, ** *p* ≤ 0.01 and depict fold changes in caspase-7 protein level in siTRIM25 versus control siRNA transfectants, which were set as one-fold.

Previously, we described the fact that the constitutive binding of the E3 ligase TRIM25 to caspase-2 mRNA represses caspase-2 translation in human colon cancer cells, thus leading to an impaired sensitivity towards drug-induced apoptosis [6]. To investigate whether the expression of other cell death regulatory proteins could be additionally influenced by TRIM25, we employed transient TRIM25 gene silencing. Based on our observations that colon carcinoma cells, upon long-term TRIM25 depletion, obviously counter regulate the resultant increase in caspase-2 by its downregulation, we deliberately chose a transient knockdown approach since other cell death-inducing proteins could be kept in check by a similar compensatory mechanism. Routinely, before the cell lysates were considered for the analysis by mass spectrometry, the knockdown of TRIM25 in biological replicates was confirmed by Western blot. The analysis of the gene-specific changes in the proteome of the human colon carcinoma cell line RKO by mass spectrometry identified a total of 226 putative TRIM25 target proteins significantly altered in TRIM25 downregulated cells (Supplementary Table S1). Of these, 140 proteins were significantly upregulated, and 86 proteins were downregulated, indicating a trend towards upregulation in TRIM25 knockdown samples (Supplementary Table S1). As the cysteine peptidase caspase-7 was the candidate that exhibited the strongest and most significant increase in expression by TRIM25 silencing (Figure 1A), it was selected for further analysis. A clear and significant increase in caspase-7 protein after different time periods of TRIM25 silencing was confirmed in four different human colon carcinoma cell lines, including RKO (Figure 1B), HCT-15 (Figure 1C), DLD-1, CaCo-2 and also in HEK293 cells (Supplementary Figure S1A,B), indicating that the inverse correlation of both proteins is not a tumor-specific phenomenon. A similar rise in caspase-7 protein was found when we employed siRNA duplexes targeting another sequence of TRIM25, supporting the finding that the increase in caspase-7 is not due to off-target effects (Supplementary Figure S1C). Conversely, the overexpression of TRIM25 was concomitant with a clear reduction in the caspase-7 levels in the RKO (Figure 1D, upper panel) and HCT-15 (Figure 1D, lower panel) cells, supporting the note that TRIM25 represents a negative modulator of caspase-7 expression.

### 3.2. TRIM25 Knockdown-Dependent Elevation of Caspase-7 Is Due to an Increase in Caspase-7 mRNA Stability

As E3 ligases play major roles in ubiquitin-mediated proteasomal degradation, we questioned whether the increase in capsase-7 upon TRIM25 silencing could result from an impaired degradation of caspase-7 protein. In that case, the increase in caspase-7 induced by TRIM25 knockdown should not be impaired by the inhibition of de novo protein synthesis. However, the administration of cycloheximide (CHX) 24 h post-transfection blunted the TRIM25 silencing-mediated rise in caspase-7 levels, thus indicating that the elevated caspase-7 contents by TRIM25 knockdown are not due to impaired caspase-7 protein degradation (Figure 1E). By contrast, the treatment of cells with the mTOR kinase inhibitor rapamycin had no effect on the caspase-7 contents, implying that the elevation in caspase-7 protein levels mainly results from cap-independent translation (Figure 1F). At the same time, the constitutive phosphorylation of p70S6 kinase was totally prevented upon rapamycin administration, which is indicative of an inhibition of mTOR kinases (Figure 1F).

**Figure 2:**
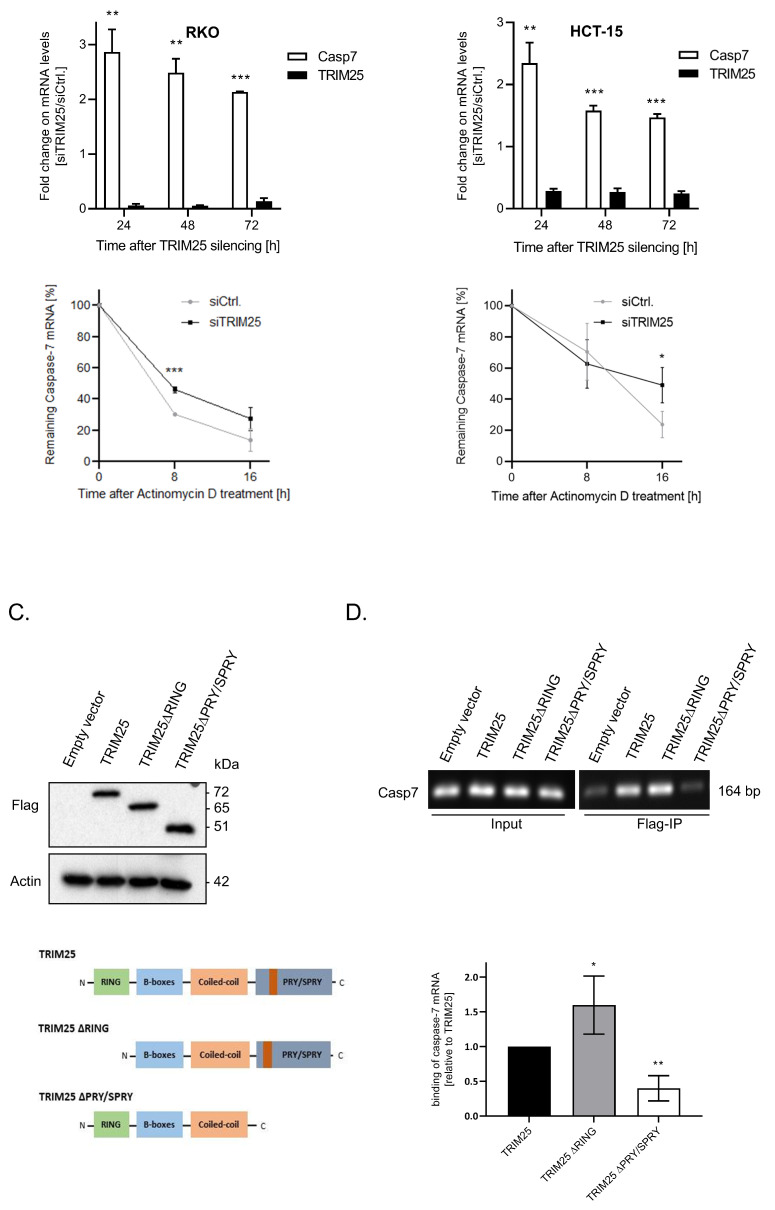
(**A**,**B**), upper panels. Time course of steady-state mRNA levels of TRIM25 and caspase-7 after siRNA-mediated TRIM25 knockdown. Steady-state TRIM25 mRNA (black bars) and caspase-7 (Casp7) mRNA levels (open bars) in RKO (**A**) or HCT-15 (**B**) cells were measured by qPCR in relation to 18S RNA and are shown as a ratio of the mRNA contents in siTRIM25 vs. control siRNA transfectants, which were set as one-fold. Data represent means ± SD (n = 3), ** *p* ≤ 0.01 and *** *p* ≤ 0.005. (**A**,**B**), lower panels. Increased stability of caspase-7 mRNA after silencing of TRIM25 in RKO (**A**) and HCT-15 (**B**) cells. Twenty-four hours after siRNA transfection, the cells were washed and subsequently treated with actinomycin D (5.0 µg/mL). Remaining mRNA contents normalized to 18S RNA at the indicated time points and compared with the levels of normalized mRNA species measured immediately before the addition of actinomycin D (0 h) and which were set as 100% are depicted for both siRNA transfectants. Data represent means ± SD (n = 3), * *p* ≤ 0.05, *** *p* ≤ 0.005. (**C**,**D**). Twenty-four hours after transfection, cells were harvested for total protein lysates, and the expression level of different Flag-TRIM25 chimeric proteins (Flag) was controlled by Western blot analysis, and β-actin was used for a control of loading equal amount of protein (**D**). RNP-IP assays from total cell lysates of RKO cells ectopically expressing TRIM25 or different TRIM25 truncations depicted in (**C**). TRIM25-bound mRNA from total cell homogenates was isolated by using anti-Flag-M2 magnetic beads followed by RT-PCR. For PCR, primer pairs, complementary and specific to the coding region of caspase-7, were used. Caspase-7 mRNA was isolated before the IP and assessed by RT-PCR using the same primer pairs (input). The lower panel summarizes data from qPCR analysis from the same RT reactions (n = 3). * *p* ≤ 0.05, ** *p* ≤ 0.01 vs. wild-type TRIM25 (TRIM25) set as one-fold. Data represent means ± SD (n = 3).

Next, we tested whether TRIM25 silencing may additionally influence the mRNA levels of caspase-7. The data from the quantitative (q)PCR revealed a robust decrease in the TRIM25 mRNA levels when compared with the control siRNA-transfected cells, concomitant with a significant increase in the steady-state caspase-7 mRNA contents independently of the time point and the cell line assessed (upper panels of Figure 2A,B). To further evaluate the impact of TRIM25 on caspase-7 mRNA decay, 24 h after the siRNA transfection the transcription in the RKO (A) or HCT-15 (B) cells was stalled by the addition of actinomycin D. Intriguingly, in both cancer cell lines, although at different particular time points after the blockade of the transcription, the remaining level of caspase-7 transcripts was significantly elevated upon TRIM25 attenuation (lower panels of Figure 2A,B), indicating that TRIM25 inhibits caspase-7 mRNA expression through a decrease in caspase-7 mRNA stability.

### 3.3. TRIM25 Binding to Caspase-7 mRNA Is Mediated through the PRY/SPRY Domain

The previous data from several laboratories report TRIM25 as an E3 ubiquitin ligase with an additional RNA-binding capacity [6,8,29,30]. To address the question of whether the negative regulation of caspase-7 expression by TRIM25 is due to its direct interaction with caspase-7 mRNA, we employed RNA immunoprecipitation (RIP). To further delineate which TRIM25 domain is involved in caspase-7 mRNA binding, we, in addition to the Flag-tagged wild-type (wt) TRIM25 (TRIM25), overexpressed two different TRIM25 truncations, which either lacked the RING domain relevant for E3 ligase activity (TRIM25 ΔRING) or was devoid of the PRY/SPRY domain (TRIM25 ΔPRY/SPRY) involved in TRIM25′s RNA-binding activity [8] in RKO cells (Figure 2C). Subsequently, the caspase-7 binding was assessed by Flag-IP. To minimize a competition between ectopic and endogenous TRIM25, these rescue experiments were performed in small hairpin (sh)TRIM25 RKO cells. We chose the clone with the strongest reduction in TRIM25 protein content (shTRIM25 #1) (Supplementary Figure S2A). Prior to this, the specific binding of the antibody and equal ectopic expression levels of the chimeric Flag-TRIM25 proteins were affirmed by Western blot (upper panel of Figure 2C). A physical interaction of TRIM25 with caspase-7 mRNA is clearly indicated by the positive PCR amplification of a cDNA from human caspase-7 mRNA (Figure 2D). The fact that TRIM25 did not coprecipitate with the caspase-7 protein indicates an exclusive protein–RNA interaction (Supplementary Figure S2B). Interestingly, the shTRIM25 RKO cells which ectopically expressed TRIM25 ΔRING retained full caspase-7 mRNA-binding affinity, which was even higher than that observed with wtTRIM25, clearly demonstrating that the E3 ligase activity of TRIM25 is not relevant for binding to caspase-7 mRNA (Figure 2D). By contrast, the affinity of Flag-tagged TRIM25 to caspase-7 mRNA was significantly impaired in cells expressing the TRIM25 ∆PRY/SPRY mutant (Figure 2D). These results are in line with previous reports highlighting the critical role of the PRY/SPRY domain in the mRNA binding of TRIM25 [8].

### 3.4. TRIM25 Interacts with hnRNPH1 through a Common Binding to Caspase-7 mRNA

**Figure 3:**
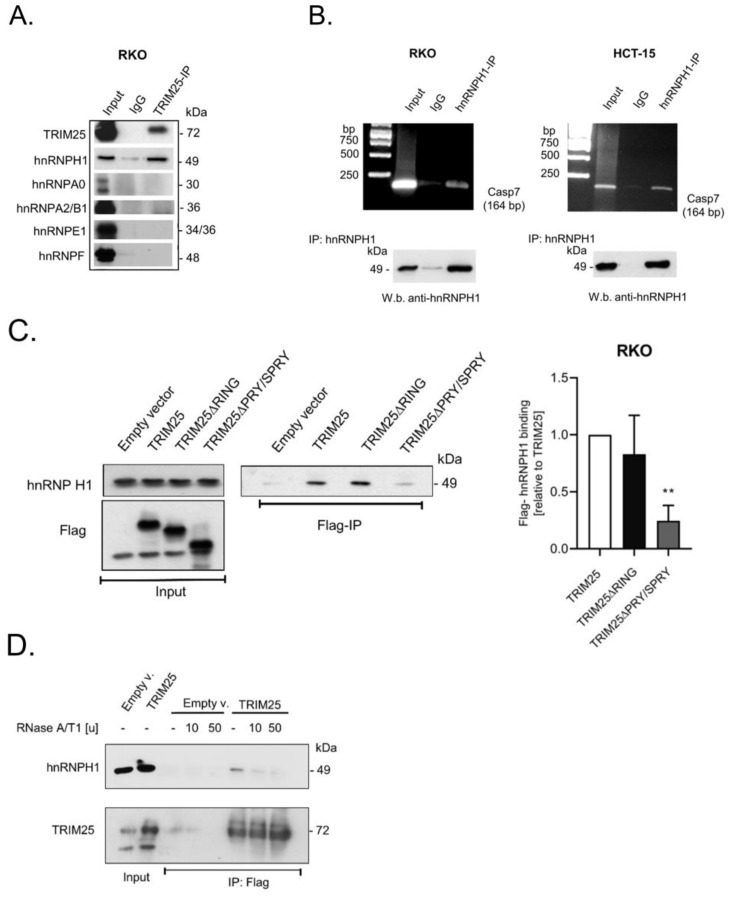
HnRNPH1 is a TRIM25-interacting and caspase-7 mRNA-binding protein. (**A**) The physical interaction of endogenous TRIM25 with the indicated members of the hnRNP family was tested after immunoprecipitation (IP) of TRIM25 (TRIM25-IP) from RKO cells or, alternatively, after treatment with isotype specific control IgG (IgG). Input levels (input) were ascertained by Western blot analysis. (**B**) RNP-IP assays from total cell lysates of RKO (left panel) or HCT-15 (right panel) cells. HnRNPH1-bound mRNA was precipitated by the addition of hnRNPH1-specific antibodies (hnRNPH1-IP) or, alternatively, the same amount of isotype specific IgG (IgG) as a negative control. RNA samples were subsequently analyzed by semiquantitative RT-PCR using primer sets encompassing the coding region of caspase-7 (Casp7) and subsequent agarose gel electrophoretic detection. The specific IP of hnRNPH1 was validated by Western blot analysis (W.b.). (**C**) shTRIM25 RKO cells were transfected with pFlag-CMV2 vector (empty), pFlag-CMV2 encoding for TRIM25 (TRIM25), or the respective deletion mutant depicted in Figure 2C. Twenty-four hours after transfection, cells were harvested for total protein lysates and the expression levels of the Flag-tagged TRIM25 mutants were monitored by Western blotting. Equal hnRNPH1 levels were also confirmed by Western blot analysis. Flag-bound hnRNPH1 from total cell homogenates was isolated by using anti-Flag-M2 magnetic beads followed by Western blot analysis with anti-hnRNPH1 antibodies. The graph shown on the right panel summarizes data from independent co-IP experiments (n = 3). ** *p* ≤ 0.01 vs. wild-type TRIM25 (TRIM25) set as one-fold. (**D**). Treatment of cell lysates with the indicated amounts of RNase A/T1 prior to the IP reaction strongly impaired the interaction of endogenous hnRNPH1 with ectopically expressed Flag-TRIM25 (TRIM25). Flag-bound hnRNPH1 from total cell homogenates was isolated by using anti-Flag-M2 magnetic beads followed by Western blot analysis with anti-hnRNPH1 antibodies. Endogenous hnRNPH1 levels were assessed by Western blot analysis (input). The IP of equal TRIM25 levels and input levels of TRIM25 were also monitored by Western blot analysis.

Employing TRIM25-IP experiments after quantitative stable isotope labeling in combination with mass spectrometry, a previous study reported that many of the candidate TRIM25 interacting proteins in HeLa cells are functionally involved in RNA metabolism. Strikingly, among the putative candidates, various members of the heterogeneous nuclear RNP (hnRNP) RBP family displayed a high affinity with TRIM25 [8]. Inspired by these data, we questioned whether members of the hnRNP family would also interact with TRIM25 in colon carcinoma cells. To this end, we employed TRIM25-IP and subsequently probed IP reactions with antibodies against different members of the hnRNP family. Interestingly, a clear co-precipitation with TRIM25 was observed for hnRNPH1 (Figure 3A). Similarly to other members of the hnRNP family, hnRNPH1 is a multifunctional protein involved in pre-mRNA splicing, mRNA trafficking, and mRNA stability [31]. Likewise, hnRNPH1 plays a critical role in several human malignancies, including colon cancer [32,33]. First, by employing RNP-IP RT-PCR experiments, we confirmed that hnRNPH1, like TRIM25, is a bona fide caspase-7 mRNA-binding protein in RKO (Figure 3B) and in HCT-15 cells (Figure 3C). Next, analogously to the RIP experiments with different TRIM25 truncations (Figure 2C), we monitored which TRIM25 domain was critical for interaction with hnRNPH1. Again, the ectopic expression of different Flag-TRIM25 chimera was performed in shTRIM25 cells to avoid a competition with endogenous TRIM25 protein. In full agreement with the results from the studies on mRNA binding (Figure 2D), the PRY/SPRY domain also seems critical for TRIM25–hnRNPH1 interactions (Figure 3C). To further analyze the impact of RNA on the TRIM25–hnRNPH1 interactions, the cell lysates were treated with RNases prior to the addition of the IP antibodies. The degradation of cellular RNA by RNase was confirmed by agarose gel electrophoresis (Supplementary Figure S3). Notably, the TRIM25 binding to hnRNPH1 was strongly compromised after the RNase treatment, indicating that the association of TRIM25 with hnRNPH1 depends on RNA (Figure 3D). The same RNase susceptibility was observed if endogenous TRIM25 instead of Flag-tagged TRIM25 was precipitated from the parental RKO cells (Supplementary Figure S3).

**Figure 4:**
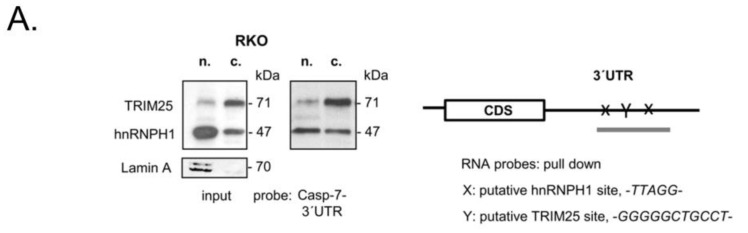

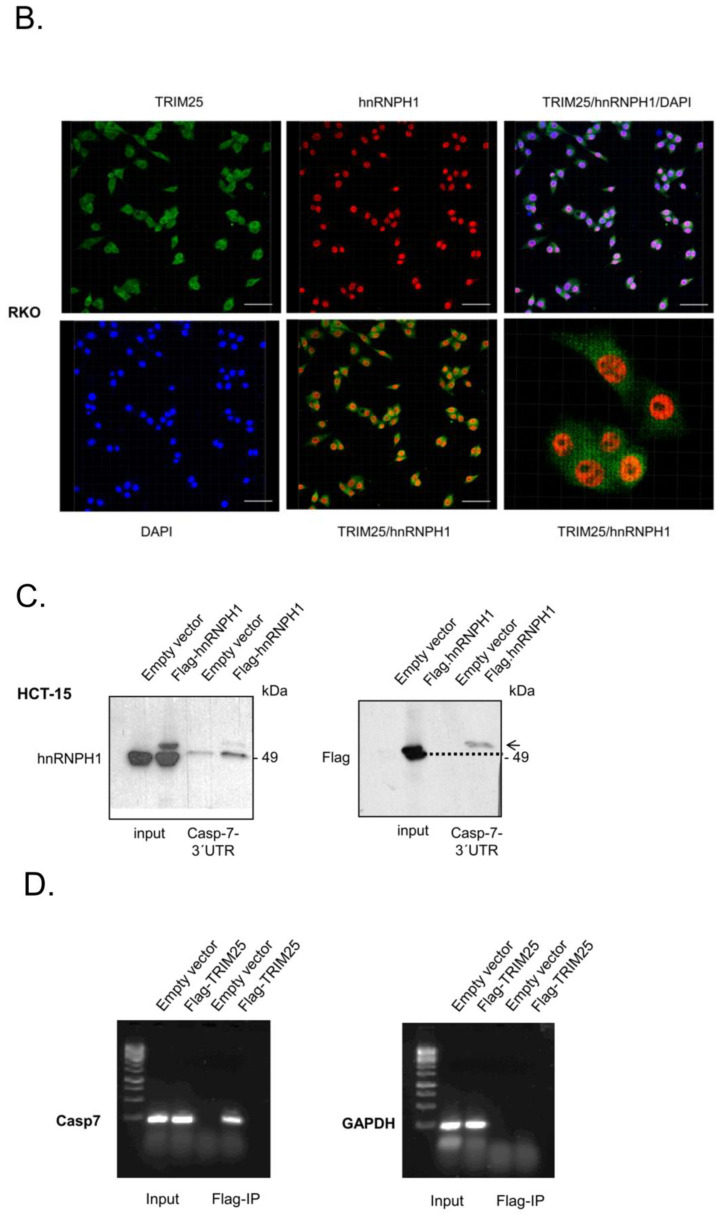
Binding of TRIM25 and hnRNPH1 to the 3′UTR of caspase-7 mRNA. (**A**) Biotin pulldown assay demonstrating different binding affinities of TRIM25 and hnRNPH1 to the 3′UTR of caspase-7. Biotinylated transcripts (20 µg) encompassing putative binding sites for hnRNPH1 (x) and TRIM25 (Y) within the 3′UTR of caspase-7 as depicted on the right panel were incubated with 300 µg of nuclear (n.) or cytoplasmic (c.) cell extracts from RKO cells. Subsequently, TRIM25 and hnRNPH1 binding to the pulldown material as well as input levels (input) was monitored by immunoblotting. Lamin A was used as a marker for cell nuclei. (**B**) Intracellular TRIM25 (green) and hnRNPH1 (red) in RKO cells were visualized by confocal microscopy. Nuclei of RKO cells were visualized with DAPI (blue). Bar: 20 µm. (**C**) Total cell homogenates derived from RKO cells either transfected with empty (empty vector) or with Flag-tagged hnRNPH1 (Flag-hnRNPH1) were analyzed for caspase-7-3′UTR binding by biotin pulldown assay. Binding of hnRNPH1 in the pulldown material and the amount of input protein were monitored by Western blot analysis using hnRNPH1-specific (left panel) or anti-Flag antibodies (right panel), respectively. (**D**) Total cell lysates derived from the same lysates were analyzed for casp-7-3′UTR binding by RNP-IP assay. Flag-bound mRNAs were precipitated by using anti-Flag antibodies (Flag-IP). Subsequently, RNA samples were analyzed by semiquantitative RT-PCR using primers encompassing the coding region of caspase-7 mRNA (left panel) or GAPDH mRNA (right panel), the latter of which was used as a negative control. Input levels of both mRNAs were monitored by RT-PCR (input).

In most cases, the control of mRNA decay in eucaryotes by RNA-binding proteins is elicited through their binding to specific cis-regulatory elements often located in the 3′ untranslated region (UTR) [34,35]. Interestingly, the 3′UTR of caspase-7 contains two sets of pentameric -UUAGG- sequences, one of prevalent binding sites described for hnRNPH [36,37]. Accordingly, the data from CLIP-seq revealed that TRIM25 preferentially binds to G- and C-rich stretches [8], although no prototypical binding sites for TRIM25 have been reported so far. RNA probe pulldown experiments with a biotinylated in vitro-transcribed RNA encompassing a putative TRIM25, as well as the two potential hnRNPH1-binding sites of the 3′UTR of caspase-7 mRNA depicted in Figure 4A, revealed a clear difference in the binding affinity of TRIM25 and hnRNPH1 to the caspase-7-specific RNA probe when different cell fractions were utilized for pulldown assays and Lamin A used a nuclear marker protein (Figure 4A). A strong binding of TRIM25 was observed with cytoplasmic fractions (c.), which is in full accordance with the divergent distribution of TRIM25, which is highest in the cytoplasm (Figure 4A). Consistently, a heterogeneous intracellular staining of TRIM25 was confirmed by immunocytochemistry (Figure 4B). Interestingly, although hnRNPH1 is predominantly localized in the nucleus (Figure 4A,B), it exerted a similar caspase-7 mRNA-binding intensity irrespective of which subcellular fraction was used for the pulldown assay (Figure 4A), implying that cytoplasmic hnRNPH1 exhibits an overall stronger binding affinity to caspase-7 mRNA than nuclear hnRNPH1. In accordance with the different nuclear cytoplasmic distribution observed after biochemical fractionation, a clear colocalization of both proteins was hard to detect by confocal microscopy (Figure 4B). Interestingly, the assessment of the RNA-binding capacity of ectopically expressed hnRNPH1 showed a clear shift in the RNA-bound chimeric Flag-hnRNPH1 protein when compared with the corresponding protein in the input control. This shift was most obvious in the blots with anti-Flag antibodies (dotted line in the right panel of Figure 4C) and indicates that caspase-7 mRNA-bound hnRNPH1 shows slightly reduced migration properties, presumably due to some posttranslational modifications. The specificity of TRIM25 binding to caspase-7 mRNA is further indicated by the results from the Flag-IP-RT-PCR analysis, demonstrating that GAPDH mRNA was not bound by ectopically expressed TRIM25 (Figure 4D).

### 3.5. Elevated Expression of Caspase-7 upon Knockdown of hnRNPH1 Is Due to an Increase in mRNA Stability

**Figure 5:**
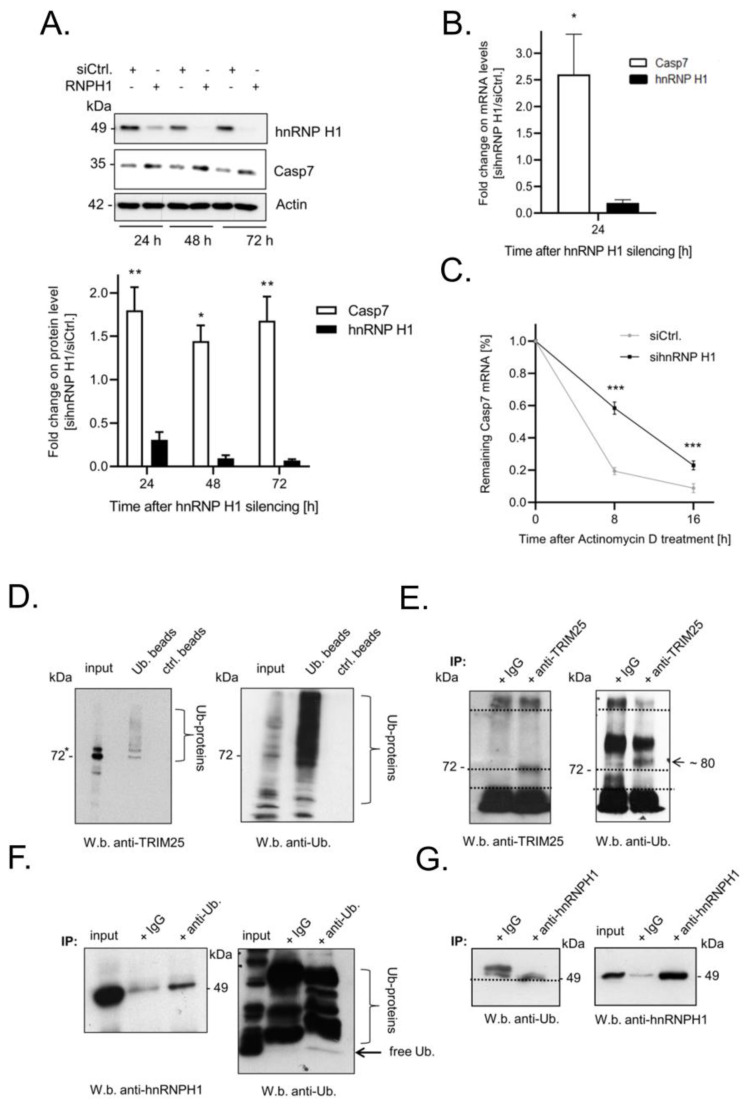
(**A**–**C**) Silencing of hnRNPH1 enhances caspase-7 expression via increasing the stability of caspase-7 mRNA. (**A**) Time-dependent changes in caspase-7 protein levels after hnRNPH1 knockdown. Subconfluent RKO cells were transfected with control siRNA duplexes (open bars) or with siRNA duplexes of hnRNPH1 (black bars) for the indicated time periods before the content of caspase-7 (open bars) was monitored by Western blot analysis. β-actin was used as a loading control. Data in the lower panel represent means ± SD (n = 3) * *p* ≤ 0.05, ** *p* ≤ 0.01 caspase-7 contents in sihnRNPH1 vs. control siRNA transfectants set as one-fold. (**B**) Steady-state mRNA levels of caspase-7 (open bar) after siRNA-mediated hnRNPH1 (black bar) knockdown were measured by quantitative real-time PCR in relation to 18S RNA levels and are shown as a ratio of the mRNA contents in sihnRNPH1 vs. control siRNA transfectants, which were set as one-fold. Data represent means ± SD (n = 3), * *p* ≤ 0.05. (**C**) Increased stability of caspase-7 mRNA after silencing of hnRNPH1 in RKO cells. Twenty-four hours after siRNA transfection, cells were washed before being treated with actinomycin D (5.0 µg/mL). The remaining mRNA contents were normalized to 18S RNA at the indicated time points and compared with the levels of normalized mRNA species measured immediately before the addition of actinomycin D (0 h) and which were set as 100%. Data represent means ± SD (n = 3), *** *p* ≤ 0.005. (**D**–**G**) Detection of ubiquitinated proteins in RKO cells by using anti-ubiquitin antibody-linked beads (Ub beads) in comparison with control beads without linked antibodies (ctrl. beads). The co-immunoprecipitation of TRIM25 was analyzed by Western blot analysis (W.b.) using a TRIM25-specific antibody. Accordingly, precipitation of ubiquitinated proteins was confirmed by immunoblotting with a monoclonal Ub-specific antibody. Input level of TRIM25 and ubiquitinated proteins were also monitored by Western blot analysis. An immunopositive TRIM25 band migrating slightly above TRIM25 is indicated by an asterisk. (**E**) Reciprocally, immunoprecipitation (IP) of TRIM25 and subsequent Western blot analysis with a polyclonal anti-Ub-specific antibody showed a concise band at 80 kDa. Dotted lines indicate the different migration properties of TRIM25 vs. ubiquitinated TRIM25. (**F**) Detection of ubiquitinated hnRNPH1 was again analyzed by using a polyclonal ubiquitin-specific antibody (anti-Ub.) in comparison with isotypic IgG (IgG). A co-IP of hnRNPH1 was confirmed by Western blot analysis using an hnRNPH1-specific antibody. IP of ubiquitinated proteins (Ub-proteins) was confirmed by Western blot with an Ub-specific antibody. Input level of hnRNPH1 and ubiquitinated proteins were monitored by immunoblotting. (**G**) Supplementarily, the IP of hnRNPH1 and subsequent immunoblotting with anti-Ub-specific antibodies revealed a concise band at 49 kDa.

Encouraged by our hypothesis that TRIM25 may indirectly affect the stability of caspase-7 mRNA via hnRNPH1, we tested whether the silencing of hnRNPH1 could mimic the effects of TRIM25 knockdown. Indeed, the RNAi-mediated knockdown of hnRNPH1 caused a significant increase in caspase-7 protein expression independently of which time point was assessed (Figure 5A, upper panel). A similar rise in caspase-7 was observed when using siRNA duplexes targeting another sequence of hnRNPH1 (Supplementary Figure S4A). An inverse correlation between hnRNPH1 and caspase-7 was confirmed in HCT-15 cells but also in HEK293 cells, which indicates that the inverse correlation between both proteins is not exclusively found in tumor cells (Supplementary Figure S4B,C). Likewise, the stable increase in caspase-7 expression by hnRNPH1 knockdown was also observed on the mRNA level (Figure 5B) and accompanied by a significant increase in caspase-7 mRNA stability (Figure 5C). Strikingly, after the stalling of transcription and the silencing of hnRNPH1, the biphasic decay of caspase-7 mRNA changed to a linear decline, suggesting that hnRNPH1 silencing did mainly affect the early phase of rapid caspase-7 degradation, a tendency which we have already observed upon TRIM25 silencing (Figure 2A). Similarly, a biphasic decay of caspase-7 mRNA was not observed in stable TRIM25 knockdown cells (Supplementary Figure S4D). Accordingly, the additional silencing of hnRNPH1 in these cells had no further effect on the stability of caspase-7 mRNA when compared to the cells transfected with the control siRNA (Supplementary Figure S4D). Together, these data suggest that TRIM25 represents a novel caspase-7 mRNA-binding protein, which, in concert with RNA-bound hnRNPH1, promotes the constitutive decay of caspase-7 mRNA in colon carcinoma cells.

### 3.6. TRIM25 and hnRNPH1 Are Both Targets of Permanent Ubiquitination

It is well documented that the RNA-related functions of TRIM25 can be accompanied by the autoubiquitination of TRIM25 [8,38]. To test whether in colon carcinoma cells TRIM25 is also a target of constitutive ubiquitination, we used ubiquitination affinity beads, which allow the capturing of ubiquitinated proteins with high affinity from whole cell lysates. The results from these experiments suggest that TRIM25 itself is a target of mono- and polyubiquitination, as indicated by the fact that above the two distinct bands either at the size of TRIM25 or slightly above, a smear of bands was observed by probing the Western blots with a TRIM25-specific antibody (Figure 5D). Conversely, a clear band at 80 kDa (the predicted size of monoubiquitinated TRIM25) was detected after the IP of TRIM25 and the subsequent Western blot with a ubiquitin-specific antibody (Figure 5E).

Next, we analyzed whether hnRNPH1 could represent a further target of ubiquitination. We observed the co-immunoprecipitation of hnRNPH1 protein upon ubiquitin-IP. Notably, a weak signal was also observed in the control reactions with IgG (Figure 5F), which can be explained by the fact that IgG itself had a certain affinity for the precipitation of hnRNPH1 protein from cell lysates (Figure 5G). Conversely, the increased abundance of an immunopositive band at 49 kDa observed upon hnRNPH1-IP in comparison to a higher migrating positive signal in IgG precipitates, possibly arising from the heavy chains of the IP antibody, further supports the notion that hnRNPH1 is a further target of ubiqutin modificaiton (Figure 5G).

### 3.7. Functional Consequences of Caspase-7 for Apoptotic Cell Death

**Figure 6:**
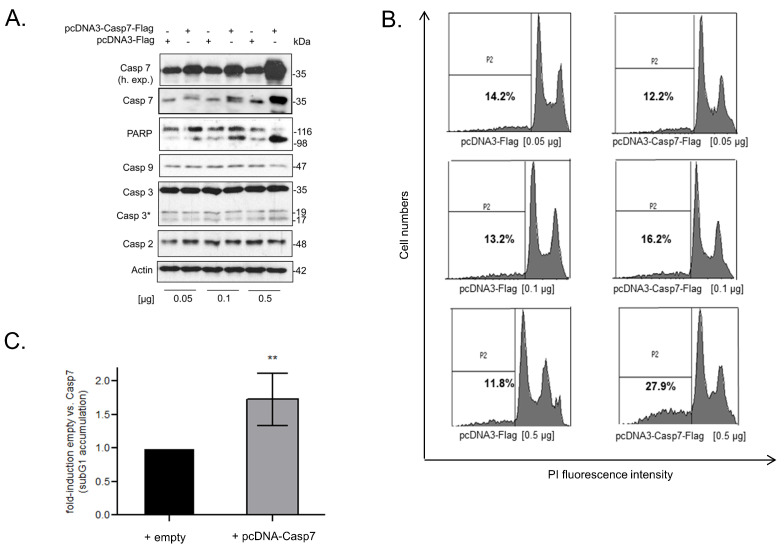

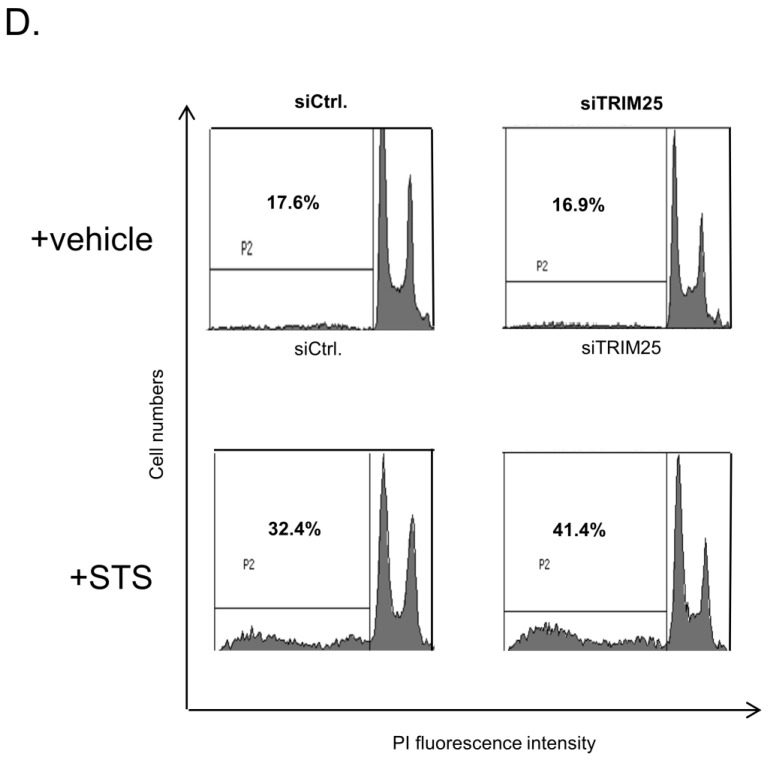

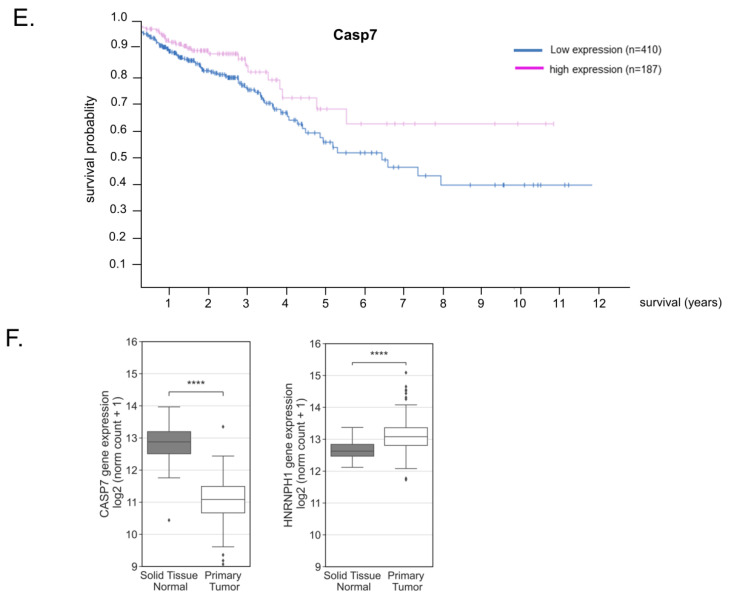
(**A**–**D**) Functional consequences of caspase-7 for apoptotic cell death. (**A**) RKO cells were either transfected with the indicated amounts of cDNA coding for human caspase-7 (pcDNA3-Casp7-Flag) or, alternatively, with the same amount of empty vector (pcDNA3-Flag). Forty-eight hours after transfection, PARP-1 cleavage and the levels of the indicated precursor caspases were monitored by Western blot analysis, and β-actin was used as a loading control. Levels of pro-caspase-7 are additionally shown at a higher exposure (h. exp.), * *p* ≤ 0.05. (**B**) Accordingly, cells were transfected with increasing amounts of pcDNA3-Casp7 (grey bar) or with the same amount of empty vector (black bar), as indicated for 48 h before being subjected to flow cytometric analysis for determination of sub-G1 accumulation (P2) by propidium iodide (PI) staining. (**C**) Values represent means ± SD (n = 3). ** *p* ≤ 0.01 from transfections with 0.5 µg pcDNA3-Casp7-Flag (+ pcDNA-Casp7) vs. empty vector (+ empty) transfectants set as one-fold. (**D**) Silencing of TRIM25 increases staurosporine (STS)-induced accumulation of colon carcinoma cells in sub-G1 phase. RKO cells were transfected with control siRNA duplexes (siCtrl.) or with siRNA duplexes of TRIM25 (siTRIM25) for 48 h before being treated for 6 h with 250 nM of STS (+STS) or with vehicle (+vehicle). Sub-G1 arrest was analyzed by flow cytometry after PI staining. Values represent means % of sub-G1 accumulation ± SD (n = 3) siTRIM25 vs. siCtrl. (**E**,**F**) Impaired expression of casaspe-7 in CRC patients correlates with reduced survival. Kaplan–Meier overall survival curves of human CRC patients with low (blue lines, n = 410) vs. high (red lines, n = 187) caspase-7 based on data from the Human Protein Atlas [40]. (**F**) Comparison of expression levels of caspase-7 and hnRNPH1 in colorectal cancer tissues (n = 288) compared with solid normal colon tissues (n = 48), analyzed with the UCSC Xena browser (htpps://xena.ucsc.edu); accessed on 22 december 2022) [41] and based on TCGA data. Non-parametric Mann–Whitney U test was used to confirm statistical significance. **** p ≤ 0.0001.

It is well established that the two executioner caspases, caspase-3 and caspase-7, are highly relevant for intrinsic apoptotic cell death [14,16]. Despite the view that both enzymes play redundant roles in apoptosis, more recent studies revealed that both proteases can exert functionally distinct roles in cell death as well as in non-apoptotic processes [15,39]. To test for the functional relevance of negative caspase-7 regulation by TRIM25 for cell death induction, we chose a gain-of-function approach by the transient overexpression of caspase-7. Importantly, the dose-dependent rise in full-length caspase-7 in RKO cells was accompanied by an increase in PARP cleavage (Figure 6A), and similar effects were observed in HCT-15 cells (Supplementary Figure S5A). Furthermore, the increase in caspase-7 was concomitant with a significant rise in apoptotic cell death indicated by the increased number of cells accumulating in sub-G1, which was most obvious after the transfection of 0.5 µg of the pcDNA-Casp7 plasmid (Figure 6B,C). Intriguingly, the basal caspase-3 cleavage in the RKO cells was not affected by the forced caspase-7 expression, indicating that the increase in PARP-1 cleavage and cell death sensitivity is mainly elicited by caspase-7 and is independent of caspase-3. Likewise, the levels of the two other caspases (caspase-2 and caspase-9) remained constant (Figure 6A), indicating that the caspase-7-mediated-death of CRC cells is not due to an increased expression of other caspases, which are generally known as upstream activators of caspase-7. This assumption is further supported by the results from the experiments with the double knockdown of TRIM25 plus caspase-2. Here, the additional knockdown of caspase-2 strongly impaired the increase in caspase-3 cleavage upon doxorubicin observed after TRIM25 silencing without affecting the cleavage of caspase-7 (Supplementary Figure S6).

### 3.8. Knockdown of TRIM25 Sensitizes Colorectal Carcinoma Cells to Drug-Induced Apoptosis

Next, we analyzed whether TRIM25 silencing could mimic the pro-apoptotic effects of caspase-7 upregulation. Therefore, the impact of TRIM25 silencing on basal and drug-induced apoptosis was tested by determination of the sub-G1 fractions. As shown in Figure 6D, the knockdown of TRIM25 had no effect on the basal sub-G1 phase cell content (Figure 6D). By contrast, the treatment with the apoptosis-inducing compound staurosporine (STS) caused a significant increase in apoptosis in TRIM25-depleted cells when compared with the control siRNA transfectants (Figure 6D). Accordingly, the silencing of TRIM25 in the RKO cells enhanced the caspase-7 cleavage by doxorubicin (doxo.) or etoposide (eto.) (Supplementary Figure S5B). Together, these data suggest that TRIM25 contributes to the resistance of colon carcinoma cells towards drug-induced cell death post-transcriptionally through suppression of caspase-7 expression.

### 3.9. Reduced Caspase-7 Expression in Primary Colonic Tumor Samples Correlates with Poor Survival

To finally evaluate whether the observed downregulation of caspase-7 in human CRC cells could be pathologically relevant for human colon carcinoma, we employed in silico analysis by using two different online tools. First, Kaplan–Meier curves obtained from the Human Protein Atlas database [40] from 597 patients clearly indicated that CRC patients with increased caspase-7 levels exhibited a higher 5-year survival (70%) than patients with low caspase-7 expression (57%, p. score 0.028) (Figure 6E). Consistently, data from the UCSC Xena platform [41], by analyzing primary tumor tissue from 288 patients versus 41 samples from noncancerous tissue, revealed a significantly decreased caspase-7 expression in primary tumors when compared to adjacent tissue (Figure 6F), confirming impaired caspase-7 expression in colorectal tumor tissue. In accordance with our hypothesis and with the published data [33], tumor tissue from CRC patients revealed an increase in hnRNPH1 expression when compared to nontumorous tissue (Figure 6F). In contrast, the expression of TRIM25 was slightly reduced in CRC tissue when compared to the solid normal tissue (data not shown). However, this is not that surprising if taking into account that TRIM25 is an E3 ligase which could be deregulated at the level of enzyme activity. Together, these data suggest a permanent downregulation of caspase-7 in CRC by the TRIM25-dependent activation of hnRNPH1, which functionally contributes to the apoptosis resistance of tumor cells.

## 4. Discussion

In this study, we report caspase-7 as a novel target of the E3 ligase TRIM25 in different human colon carcinoma cell lines and in HEK293 cells. The results from the loss- and gain-of-function experiments imply that TRIM25 exerts a constitutive suppressive effect on caspase-7 expression mainly through destabilization of caspase-7 mRNA and thereby contributes to the primary drug resistance of CRC cells. TRIM25 represents a group of E3 ligases of RNA-binding properties recently discovered by high-throughput proteome analysis [42], supporting the concept that key enzymes of proteasomal degradation and protein modification can additionally influence diverse RNA functions [43]. Functionally, the overexpression of caspase-7 caused a significant increase in PARP cleavage concomitant with an increase in sub-G1 phase cells (Figure 6A–C). Notably, the ectopic expression of caspase-7 was not accompanied by an increase in caspase-3 cleavage, which indicates that caspase-7 is acting independently of caspase-3. These results further support the notion that both executioner caspases can exert non-redundant functions in cell death processes [15,16]. Consistently, the silencing of TRIM25 increased the sensitivity to drug-induced apoptosis (Figure 6D, Supplementary Figure S5B). Searching for a possible clinicopathological relevance of the observed negative regulation of caspase-7, the data from the Xena platform [41] showed a significant decrease in caspase-7 mRNA expression levels in the primary tumor tissue from CRC patients when compared to normal adjacent tissue. Accordingly, the survival in patients with low caspase-7 levels is diminished (Figure 6E). This is in accordance with reports which have demonstrated a downregulation of caspase-7 in CRC [17,44]. In contrast, a consistent overexpression of caspase-7 has been observed in breast cancer where caspase-7 levels correlated with the estrogen receptor status of the disease but did not correlate with the rate of apoptosis, suggesting that cell death regulation is not relevant for the reported tumorigenic role of caspase-7 in breast cancer [45]. The negative posttranscriptional gene regulation of caspase-7 by TRIM25 found here may at least partially account for the impaired caspase-7 expression in colon cancer. Apart from its apoptotic functions, some non-apoptotic functions have been attributed to this executioner caspase. In this, the observed negative regulation by TRIM25 may have an additional impact on other caspase-7-triggered events, such as inflammation [16]. In the context of inflammation, previous data have unraveled caspase-7 as a direct substrate of caspase-1, which is indispensable for PARP-1 cleavage during pyroptosis [46].

With regard to TRIM25, its oncogenic potential has been mainly assigned to the modulation of diverse oncogenic signaling pathways, including the Keap1-Nrf2 pathway, which is relevant for liver cancer [47]; the p53-G3BP2 interactions, which are critical for prostate cancer cell growth [48]; or the stabilization of the enhancer of the zeste 2 homologue subunit 2 (EZH2), a critical histone methyl-transferase and epigenetic silencer complex which is relevant for the oxaliplatin resistance of colorectal cancer [49]. In this regard, our data complement the large repertoire of transcriptional and posttranscriptional events controlled by TRIM25.

Structurally, we found that the PRY/SPRY domain is also indispensable for TRIM25-caspase-7 mRNA binding (Figure 2D), which is in full accordance with the previous data [8]. In contrast, the lack of the RING domain had no effects on TRIM25s mRNA binding, indicating that the ubiquitin ligase activity is not required for RNA binding. Conversely, the RNA binding of TRIM25 was found as a prerequisite for its ubiquitin ligase activity towards the zinc finger antiviral protein ZAP [50]. The mentioned study demonstrated that TRIM25, in addition to its well-documented functions in interferon signaling, can modulate diverse RNA processing events via posttranslational modifications of adjacent RNA-binding proteins. We suggest that in a similar way TRIM25 could use caspase-7 mRNA as a scaffold to come into close contact with neighboring RNA-binding proteins as shown here for hnRNPH1. In keeping with our data, a previous report demonstrated that TRIM25 interferes with influenza A infection via destabilizing viral mRNA [51]. Our finding is furthermore in accordance with data from hnRNPH1-IP transcriptome analysis identifying caspase-7 as a putative RNA target of hnRNPH1 in the colorectal cancer cell line HCT116 [33]. The authors of this particular study postulated that hnRNPH1 increased colorectal cancer cell progress by inhibiting apoptosis mainly through the binding and stabilization of sphingosine 1-phosphate lyase (SGPL1) mRNA, an enzyme which is relevant for the degradation of the ubiquitous lipid signal mediator sphingosine 1-phosphate. Furthermore, the authors of the study found that hnRNPH1 did not influence SGPL1 expression in non-tumorous cells, implying that the hnRNPH1 regulation of target mRNA is rather a tumor-specific phenomenon. Their data underline the oncogenic potential of hnRNPH1 reported in different human cancers [52,53], including colorectal carcinoma [32]. Concordantly, the aberrant expression of hnRNPH1 in colorectal tumor tissue is also evident in the data from the USC Xena platform (Figure 6F).

In keeping with our findings, a study conducted by employing quantitative SILAC-based co-IP assays has identified various members of the hnRNP protein family as potential TRIM25-interacting proteins, supporting the note that TRIM25 may act in concert with other RBPs to control diverse mRNA functions [8]. Whereas mechanisms underlying the control of alternative splicing by different hnRNP proteins are well documented [53,54,55], the modulation of mRNA stability by hnRNPH1 is only poorly understood. The retention of introns with premature termination codes is one previously described mode of attenuation of pre-mRNA stability by hnRNPH1 [56]. Presumably, the control of the different effects of hnRNPH1 on mRNA decay may additionally depend on diverse posttranslational modifications (PTMs), including phosphorylation, methylation, SUMOylation and ubiquitination, as exemplarily described for hnRNPK [57]. Along with this, we revealed that hnRNPH1 is a putative target of ubiquitination (Figure 5F,G). Further studies are needed to prove that the ubiquitination of hnRNPH1 observed here is functionally relevant for its impact on caspase-7 mRNA stability. Notably, the co-immunoprecipitation of TRIM25 with hnRNPH1 was impeded by the RNase treatment. In full accordance with this, the truncation of the PRY/SPRY domain, which has been previously identified as a critical RNA-binding domain of TRIM25 [8], did not only impair TRIM25-caspase-7 mRNA binding but also impeded its physical interaction with hnRNPH1 (Figure 3C). Together with the results from RIP, these findings support our model that both TRIM25 and hnRNPH1 interact mainly through their common binding to caspase-7 mRNA. In agreement, TRIM25 and hnRNPH1 showed a strong binding to an RNA probe from the 3′UTR of caspase-7 mRNA, bearing putative binding sites for both RBPs (Figure 4A). However, with respect to their different intracellular distribution (Figure 4B), we suggest that the 3′UTR of caspase-7 acts as a common platform for both proteins, with the RNA being required for the modulation of TRIM25’s ubiquitin ligase activity on hnRNPH1 in a similar way to that previously reported for the RNA-binding protein ZAP, a TRIM25 target relevant for the suppression of HIV-1 replication [50]. Whether TRIM25 could similarly utilize caspase-7 mRNA as a scaffold to mediate the ubiquitination of hnRNPH1 in a way that has been described for other E3 ubiquitin ligases [58] is a subject of ongoing research in our laboratory.

## 5. Conclusions

In summary, our data identified a posttranscriptional TRIM25-hnRNPH1-caspase-7 axis as a novel pathway potentially contributing to the apoptosis resistance of CRC cells towards pharmacological stressors and highlighted TRIM25 inhibitory strategies as a novel strategy for CRC management in clinical practice.

## Data Availability

The data presented in this study are available on request from the corresponding author. The mass spectrometry proteomics data have been deposited in the ProteomeXchange Consortium via the PRIDE [60] partner repository with the dataset identifier PXD037182.

## Supplementary Materials

The following supporting information can be downloaded at: https://www.mdpi.com/article/10.3390/cells12010201/s1, Figure S1: (a) effects of TRIM25 silencing on caspase-7 protein contents in DLD-1 and CaCo-2 cells, (b) effects of TRIM25 silencing on caspase-7 protein contents in HEK293 cells, (c) upregulation of caspase-7 by two different TRIM25-specific siRNAs, Figure S2: (a) knockdown efficiency of different shTRIM25 RKO cell clones, (b) TRIM25 does not physically interact with caspase-7 protein, Figure S3: HnRNPH1 binding to TRIM25 is impaired after RNase treatment, Figure S4: (a) upregulation of caspase-7 by two different hnRNPH1-specific siRNAs, (b) knockdown of hnRNPH1 increases caspaspe-7 levels in HCT-15 cells and HEK 293 cells (c,d) silencing of hnRNPH1 has no effect on caspase-7 mRNA stability in stable shTRIM25 RKO cells, Figure S5: (a) ectopic expression of caspase-7 induces PARP1 cleavage in RKO cells, (b) knockdown of TRIM25 enhances the chemotherapeutic drug-induced caspase-7 cleavage in RKO cells, Figure S6: TRIM25 silencing dependent cleavage of caspase-7 upon doxorubicin, in contrast to caspase-3, is not affected by additional knockdown of caspase-2, Table S1: LC/MS analysis showing a list of genes significantly modulated upon siRNA-mediated TRIM25 knockdown [59].
