# Supplementary material for: The E3 Ligase TRIM25 Impairs Apoptotic Cell Death in Colon Carcinoma Cells via Destabilization of Caspase-7 mRNA: A Possible Role of hnRNPH1
Source: Cells. 2023 Jan 3;12(1):201. doi: 10.3390/cells12010201 (PMC9818768; doi:10.3390/cells12010201)
Supplement: Supplementary file 1 [file cells-12-00201-s001.zip › cells-1984012-supplementary.pdf]

Supplementary information

Supplementary Materials and Methods

The following antibodies were used in this study:

Polyclonal anti-TRIM25 (# 115737) and anti-hnRNPH1 (ab 10374) were from from Abcam (Berlin, Germany). A polyclonal antibody against ubiquitin (#BML-PW8810) was from Enzo Lifesciences (Lörrach, Germany).

Anti-caspase-3 (# 9662), anti-caspase-7 (# 9494), anti-caspase-9 (# 9508), anti-phospho-S6 ribosomal protein (Ser240/244,# 2215), anti-PARP-1 (# 9542) and anti-hnRNPE1 (# 8534) were from Cell Signaling and monoclonal anti-TRIM25 (sc-166926), mouse anti-ubiquitin (sc-8017), anti-hnRNPA0 (sc-16509), anti-hnRNPA2/B1 (sc-80993), and anti-hnRNPF (sc-32309) from Santa Cruz Biotechnology (Heidelberg, Germany). The anti-caspase-2 (# 611022) antibody and was from BD Biosciences (Heidelberg, Germany), anti- -actin antibody (A 2228), anti-hnRNPH1 from mouse and (anti-Flag antibody (F 1804) and anti-Flag-M2 magnetic beads (M 8823) were from Merck (Darmstadt, Germany) and the monoclonal anti-hnRNPH1 antibody (#MA5-27369) was from ThermoScientific (Dreieich, Germany). The goat anti-rabbit (sc-2054) and goat anti-mouse (sc-20559) HRP-linked antibodies were from Santa Cruz Biotechnology and anti-mouse DyLight488-coupled and anti-rabbit DyLight550-coupled secondary antibodies were from ThemoFisher Scientific.

Mass spectrometry (LC-MS)

Fractions were shot as described previously [59]. Basically the peptides were separated on an Easy nLC 1200 (ThermoFisher Scientific) and 1µg of peptides were eluted by a non-linear gradient adapted for each fraction over 150 minutes followed by a step-wise increase to 75% ACN in 6 minutes which was held for another 9 minutes. Peptides then directly sprayed into

an Orbitrap Fusion Lumos mass spectrometer equipped with a nanoFlex ion source (ThermoFisher Scientific).

Full scan MS spectra (350-1400 m/z) were acquired with a resolution of 120,000 at m/z. The most intense precursors with a charge state between 2 and 5 per full scan were selected for fragmentation. MS2 scans were performed in the Ion trap (Turbo) and fragmented using CID with a normalized collision energy of 35%. SPS-MS3 scans for quantification were performed on the 10 most intense MS2 fragment ions with an isolation window of 0.7 Th (MS) and 2 m/z (MS2). Ions were fragmented using HCD with an NCE of 65% and analyzed in the Orbitrap with a resolution of 50,000 at m/z 200.

Raw data was analyzed with Proteome Discoverer 2.4 (ThermoFisher Scientific). SequenceHT node was selected for database searches. Human trypsin digested proteome (Homo sapiens SwissProt database (TaxID:9606, version 12 March 2020)) was used for protein identifications. TMT6 (+229.163) at the N-terminus, TMT6 (K, +229.163) at lysine and carbamidomethyl (+57.021), and cysteine residues were set as fixed modifications. Methionine oxidation (M, +15.995) and acetylation (+42.011) at the protein N-terminus were set for dynamic modifications. Normalized abundances from protein file were used for statistical analysis after contaminations and complete empty values were removed. Significantly altered proteins were determined by a two-sided, unpaired Student's t-tests (p-value < 0.05), adding minimum fold-change cut-off (≥ 0.5) with R version 4.0.2

Legends for Supplementary Table and Figures

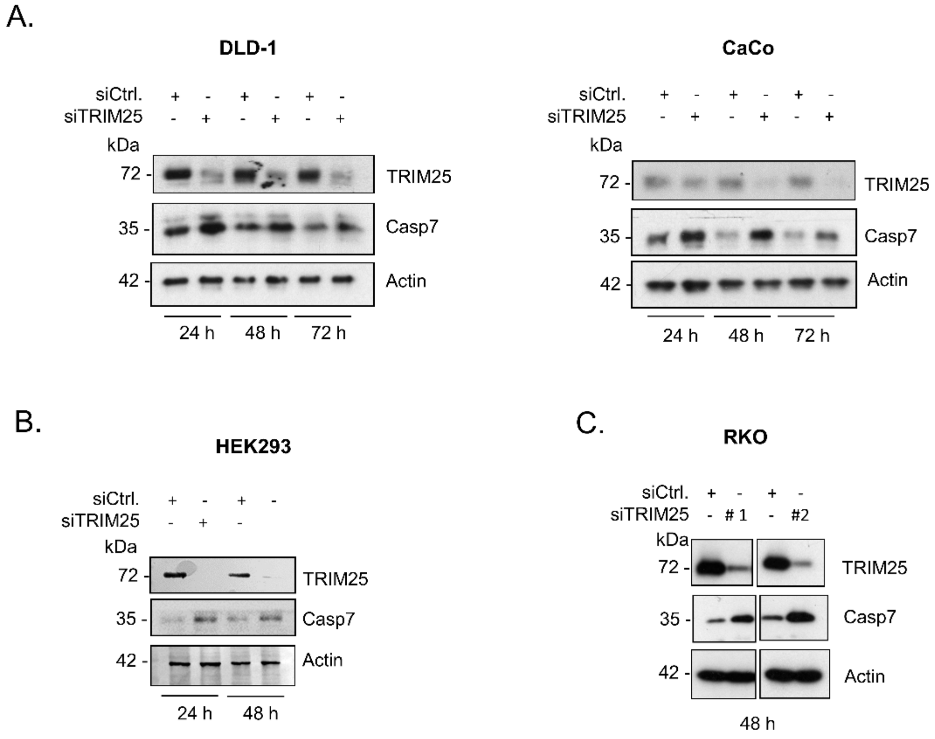

Supplementary Figure S1. *(A).* Time-dependent changes in caspase-7 protein levels after TRIM25 knockdown in DLD-1 and CaCo cells. Subconfluent DLD-1 (left panel) or CaCo (right panel) cells were transfected with control siRNA duplexes (siCtrl.) or with siRNA duplexes of

TRIM25 (siTRIM25) for the indicated time periods before the content of capase-7 was monitored by western blot analysis. -actin (Actin) was used as loading control. *(B).* In a similar way, HEK293 cells were transfected with control siRNA duplexes (siCtrl.) or with siRNA duplexes of TRIM25 (siTRIM25) for the indicated time periods before the content of capase-7 was monitored by western blot analysis. -actin (Actin) was used as loading control. *(C).* Elevations in caspase-7 levels upon TRIM25 knockdown in RKO cells were analyzed 48 h after siRNA transfection and validated by using two different sets of siRNA duplexes complementary to distinct regions of the TRIM25 mRNA (#1 and #2) as described in the Materials and Methods section.

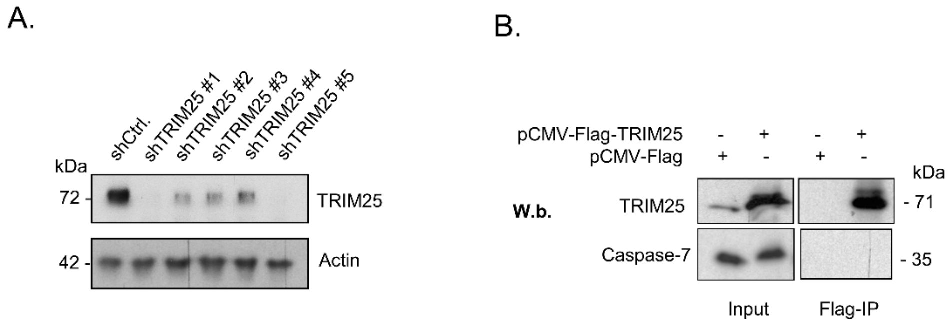

Supplementary Figure S2. *(A).* Immunoblot of TRIM25 leves in shcontrol (shCtrl.) RKO cells and different clones from shTRIM25 knockdown RKO cells obtained by lentiviral transduction of different TRIM25 specific shRNA plasmids (shTRIM25#1-shTRIM25#5). Staining of -actin (Actin) was used for control of equal loading. Target sequences of pLenti shRNA plasmids

(Sigma) are described in the Materials and Methods section. *(B).* Western blot analysis after immunoprecipitation of Flag-tagged TRIM25 (Flag-IP) in RKO cells demonstrating that TRIM25 does not interact with caspase-7 protein. RKO cells were transfected with pFlag-CMV2 encoding TRIM25 (pCMV2-Flag-TRIM25) or with with pFlag-CMV2 empty vector (pCMV-Flag). 48 h after transfection, cells were harvested for total protein lysates and Flag-tagged proteins were isolated by using anti-Flag-M2 magnetic beads followed by westernblot analysis with either TRIM25 or caspase-7-specific antibodies. Equal level of input caspase-7 were confirmed by western blot analysis.

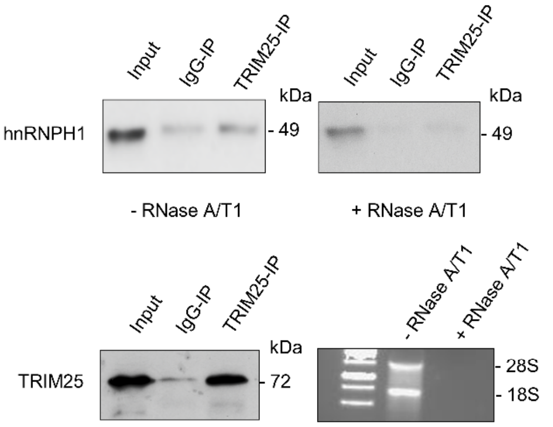

Supplementary Figure S3. Immunoblot of hnRNPH1 after IP of endogeneous TRIM25 (TRIM25-IP) in comparison to treatment with isotype specific control IgG (IgG). Input levels (Input) of both proteins and the specific IP of TRIM25 was validated by western blot analysis (lower left panel). Treatment of cell lysates with 10 u of RNase A/T1 (+RNase A/T1) prior to

the IP reaction strongly impaired the co-IP of hnRNPH1. The degradation of RNA after RNase treatment was confirmed by agarose gel electrophoresis (lower right panel).

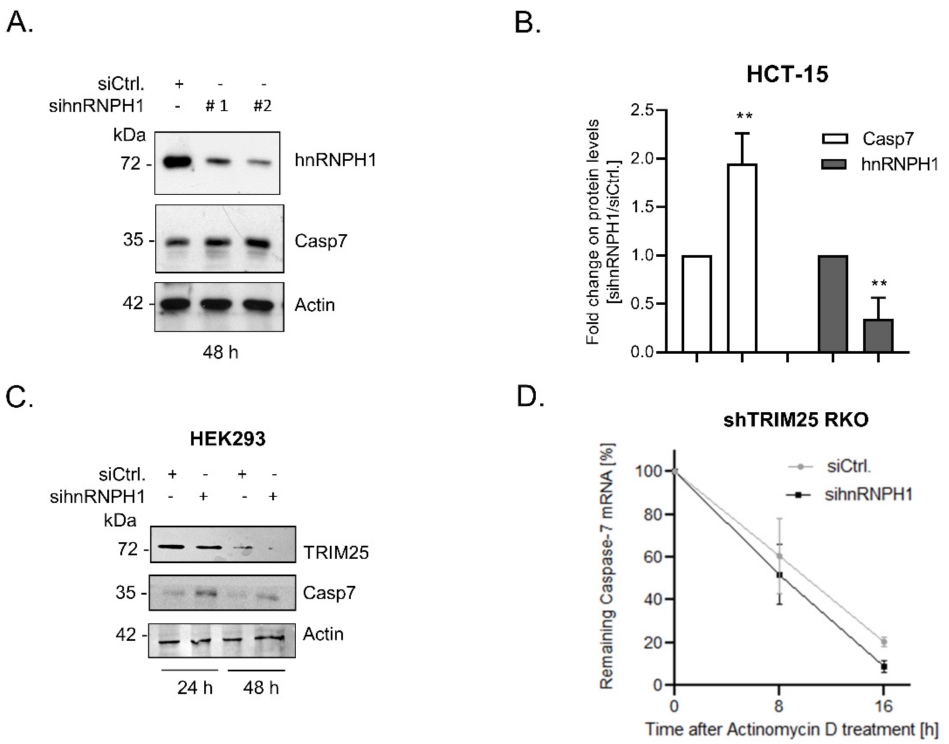

Supplementary Figure S4. *(A).* Increased caspase-7 levels upon hnRNPH1 knockdown in RKO cells were analyzed 48 h after siRNA transfection and validated by two sets of siRNA duplexes complementary to distinct regions of the hnRNPH1 mRNA (#1 and #2) described in materials and methods. *(B).* Silencing of hnRNPH1 leads to an increased content of caspase-7 protein. Subconfluent RKO cells were transfected with control siRNA duplexes (siCtrl.) or with siRNA duplexes of hnRNPH1 (sihnRNPH1) for 48 h before the contents of capase-7 (open bars) and hnRNPH1 (black bars) were monitored by western blot analysis and normalized to -actin (Actin) levels. Data represent means ± SD (n=3** $p \leq 0.01$ contents in caspase-7 or hnRNPH1 in sihnRNPH1 samples vs. control-siRNA transfectants set as one-fold. *(C).* Time-dependent changes in caspase-7 protein levels after TRIM25 knockdown in HEK293 cells. Cells were transfected with control siRNA duplexes (siCtrl.) or with siRNA duplexes of TRIM25 (siTRIM25) for the indicated time periods before the content of capase-7 was monitored by

western blot analysis. -actin (Actin) was used as loading control. *(D).* Silencing of hnRNPH1 does not affect stability of caspase-7 mRNA in shTRIM25 RKO cells. 24 h after siRNA transfection (siCtrl. or sihnRNPH1), cells were washed and subsequently exposed to actinomycin D (5.0 μg/ml). Remaining mRNA contents normalized to 18S RNA at the indicated time points and compared with the levels of normalized mRNA species measured immediately before the addition of actinomycin D (0 h) and which were set as 100% are depicted for both siRNA transfectants. Data shown are respresentative for two independent experiments.

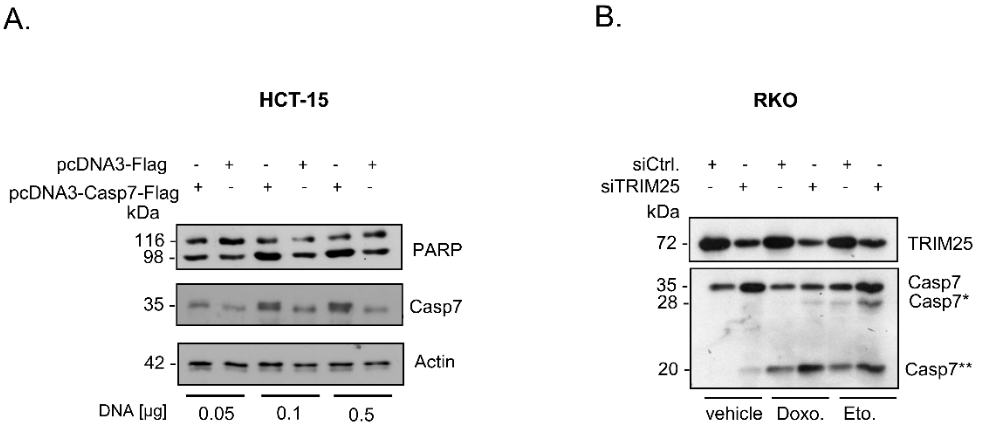

Supplementary Figure S5. (A). HCT cells were either transfected with the indicated amounts of DNA coding for human caspase-7 (pcDNA3-Casp7-Flag) or, alternatively, with the same amount of empty vector (pcDNA3-Flag). 48 h after transfection, PARP-1 cleavage and forced expression of caspase-7 was monitored by western blot analysis and -actin (Actin) was used as a loading control. (B). RKO cells were either transfected with control siRNA (siCtrl.) or with siRNA duplexes against TRIM25 (siTRIM25) for 24 h before cells were exposed to doxorubicin

(Doxo.) or etoposide (Eto.) for 16 h. Thereafter, cells were lyzed and total homogenates analyzed for caspase-7 cleavage and TRIM25 was validated by westernblot analysis. Different cleavage products of caspase-7 are indicated by asterisks and double asterisks.

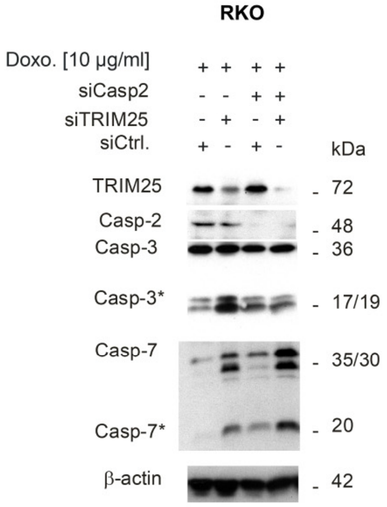

Supplementary Figure S6. RKO cells were transfected with control siRNA (siCtrl.) or with siRNA duplexes either targeting caspase-2 (siCasp2) or TRIM25 (siTRIM25) or, alternatively, simultaneously transfected with TRIM25 plus caspase-2-specific siRNAs for 24 hours before cells were exposed to doxorubicin for further 24 hours. Thereafter, cells were collected for total protein lysis and activation of caspase-7 cleavage and knockdown efficiency of TRIM25 or caspase-2 determined by Western blot using β-actin as a control for equal protein loading.

Supplementary Table S1.

List of genes significantly upregulated (fold changes ≥ 0.5, highlighted in red), or downregulated (fold changes ≤ -0.5, highlighted in green) upon siRNA-mediated TRIM25 knockdown (n=3) as derived from LC/MS analysis.
